# Association of Mediterranean-DASH Intervention for Neurodegenerative Delay (MIND) diet and metabolically unhealthy overweight/obesity phenotypes among Iranian women: a cross sectional study

**DOI:** 10.1186/s12902-023-01333-2

**Published:** 2023-04-19

**Authors:** Alireza Khadem, Farideh Shiraseb, Atieh Mirzababaei, Sahar Noori, Khadijeh Mirzaei

**Affiliations:** 1 Department of Nutrition, Science and Research Branch, Islamic Azad University, Tehran, Iran; 2 Department of Community Nutrition, School of Nutritional Sciences and Dietetics, Tehran University of Medical Sciences (TUMS), Tehran, Iran; 3 Food Microbiology Research Center, Tehran University of Medical Sciences, Tehran, Iran

**Keywords:** Metabolic healthy, Metabolic unhealthy, MIND diet, Obesity and overweight

## Abstract

**Purpose:**

Paradoxes have been found in obesity, including individuals with metabolically healthy obesity (MHO) and metabolically unhealthy obesity (MUHO), and diet may be one of the reasons for the creation of these metabolic phenotypes. Hence, the purpose of the present study was to investigate the association of the Mediterranean-DASH intervention for neurodegenerative delay (MIND) diet with metabolically unhealthy overweight/obesity (MUHOW/O) phenotypes.

**Methods:**

In this cross-sectional study, 229 overweight and obese women (body mass index (BMI) ≥ 25 kg/m2) aged 18–48 years were examined. Anthropometric measures and biochemical parameters were collected from all participants. The body composition of each participant was assessed using a bioelectrical impedance analyzer (BIA). The MIND diet score was determined based on 15 components using a valid and reliable food frequency questionnaire (FFQ) containing 147 items. Karelis criteria was used to determine metabolically healthy/unhealthy phenotype (MH/MUH).

**Results:**

Among the participants, 72.5% of individuals were identified as MUH and 27.5% as MH, with a mean ± standard deviation (SD) age of 36.16 (8.33) years. The results of our analysis showed that after controlling for age, energy intake, BMI, and physical activity, there was no significant association observed between overweight/obesity phenotypes with tertile 2 (T2) (OR: 2.01, 95% CI: 0.86–4.17, P-value = 0.10), T3 (OR: 1.89, 95% CI: 0.86–4.17, P-value = 0.11) of MIND score, and only the odds of MUH relative to MH with a marginal significant decreasing trend was observed from the second to the third tertile (1.89 vs. 2.01) (P − trend = 0.06). Also, after additional adjustment for marital status, the nonsignificant association between overweight/obesity phenotypes with tertile 2 (T2) (OR: 2.13, 95% CI: 0.89−5.10, P-value = 0.08), T3 (OR: 1.87, 95% CI: 0.83−4.23, P-value = 0.12) of MIND score remained, and the odds of MUH relative to MH with a significant decreasing trend was observed with increasing tertiles (P-trend = 0.04).

**Conclusions:**

In conclusion, no significant associations were found between adherence to MIND diet with MUH, and only a significant downward trend in the odds of MUH was observed with increasing tertiles. We suggest further studies in this field.

## Introduction

Obesity is considered a major public health problem and has a high prevalence in both developing and developed countries [1–3]. In Iran, the prevalence of obesity is reported to be 26.1% in adults [4], which was different between men and women, so that women were more prevalent than men [5]. This higher prevalence of obesity in women can be due to less physical activity, physiological differences in the composition and distribution of adipose tissue, hormonal factors, and some neurological diseases such as depression [6]. Obesity is associated with an increased risk of diseases such as type 2 diabetes (T2D), cardiovascular diseases (CVDs), and various types of cancer, as well as metabolic disorders [7, 8].

Recently, two obesity-related phenotypes, namely metabolically healthy obesity (MHO) and metabolically unhealthy obesity (MUHO), have attracted much attention [9, 10]. People with healthier phenotypes are often younger and more physically active, and despite having a body mass index (BMI) ≥ 30 kg/m2, they have better lipid profiles, higher levels of insulin sensitivity, and a lower risk of CVDs compared to obese people who are metabolically unhealthy [11, 12]. Various criteria are used to determine the MHO [13]. One of these indicators is the Karelis criteria, which examines inflammatory profiles, insulin sensitivity, and parameters related to the lipid profile [14]. Studies have shown that these metabolic phenotypes of obesity may be due to genetic and environmental factors as well as the interactions between them [15]. Including environmental factors, diet can be referred to as one of the main factors that could affect the creation of obesity phenotypes [16].

Diet is a modifiable risk factor for obesity. Previous studies have investigated the relationship between dietary patterns and obesity phenotypes [12, 17–19]. Among the dietary patterns that can be mentioned are the Dietary Approaches to Stop Hypertension (DASH) diet and the Mediterranean dietary pattern (MD), which in previous studies showed a favourable relationship with metabolically healthy phenotypes [18, 20, 21]. However, there were conflicting findings [12, 22–24]. Although adherence to the DASH diet is inversely related to decreased high-density lipoprotein cholesterol (HDL-C) levels and elevated triglyceride (TG) levels [25], and adherence to the MD diet is associated with decreased insulin resistance (IR) and inflammation [26], these associations were not found in other studies [27, 28]. Recently, a combination of these two dietary patterns, the DASH and Mediterranean diets, called the Mediterranean-DASH intervention for neurodegenerative delay (MIND) diet, has been proposed [29]. The MIND diet is based on healthy and unhealthy brain foods and consists of 15 components, 10 of which are brain-healthy foods and 5 of which are brain-unhealthy foods [29]. The MIND diet emphasises the consumption of berries, which, due to their abundant phenolic compounds, may be effective in improving several metabolic abnormalities such as lipid profiles [30]. Also, due to the components of this diet, which emphasises natural plant foods, high fiber, and a low glycemic index, it may be effective in improving the lipid profile and regulating blood sugar [31]. On the other hand, this diet contains foods such as ready-made foods, fried foods, butter, margarine, and sweets that are not included in the MD and DASH diets.

Although the relationship between DASH and MD diets and obesity phenotypes has been separately assessed, this study aims to investigate the association between the MIND diet and metabolically unhealthy overweight/obesity phenotypes among Iranian women.

## Methods and materials

In this cross-sectional study, 229 women referring to health centers and nutrition clinics in Tehran were examined. Inclusion criteria for people with BMI ≥ 25 kg/m^2^ and aged 18–48 years. Exclusion criteria included any history of disease, including CVDs, diabetes, cancer, kidney disease, and thyroid disease, as well as menopause, pregnancy, and lactation, and reporting a total daily energy intake < 800 kcal/d and 4200 kcal/d<. In addition, individuals taking drugs that lower lipids, blood glucose, or blood pressure were excluded. Participants and legal guardians for illiterate participants were fully informed about the study protocol and provided written and informed consent. The study protocol was approved by the ethics committee of Tehran University of Medical Sciences (TUMS) with the following identification: IR.TUMS.MEDICINE.REC.1400.710.

### Measurement of biochemical parameters

Blood samples were obtained between 8:00 and 10:00 a.m. following overnight fasting at the Nutrition and Biochemistry Laboratory of the School of Nutritional Sciences and Dietetics, TUMS, and collected into tubes containing 0.1% ethylenediaminetetraacetic acid (EDTA). The serum was centrifuged, aliquoted, and stored at − 70 °C. Fasting blood sugar (FBS) was measured using the GOD/PAP (glucose oxidase, phenol, 4-aminoantipyrine peroxidase) method. Glycerol-3-phosphate oxidase Phenol 4-Aminoantipyrine peroxidase (GPO-PAP) enzymatic colorimetric tests were used to determine serum TG concentrations. Total cholesterol (total chol) levels were measured by the cholesterol oxidase Phenol 4-Aminoantipyrine Peroxidase (CHOD-PAP), and low-density lipoprotein (LDL) and HDL were measured by the direct method and immunoinhibition. Serum hypersensitive C-reactive protein (hs-CRP) was measured by an immunoturbidimetric assay. All kits were from Pars Azmoon (Pars Azmoon Inc., Tehran, Iran). Serum insulin concentrations were analysed through the enzyme-linked immunosorbent assay (ELISA) method [12].

### Assessment of anthropometric measures

Weight was determined with digital scales while the subjects were minimally clothed and without shoes and recorded to the nearest 100 g. Height was measured using a tape measure while participants were in the normal standing position, without shoes. BMI was calculated by dividing the weight (kg) by the square of the height (m^2^).

### Assessment of blood pressure

After 10–15 min of rest, blood pressure measurements were taken using a standard mercury sphygmomanometer (Omron, Germany, European).

### The HOMA-IR calculation

The insulin resistance homeostatic model assessment (HOMA-IR) was determined based on the following equation: HOMA-IR = [fasting plasma glucose (mmol/l) × fasting plasma insulin (mIU/l)] /22.5 [32].

### Definition of metabolic health and its components

We used Karelis criteria, according to which the presence of 4 or more of the following items indicates a metabolically healthy (MH) phenotype: TG ≤ 1.7 mmol/l, HDL ≥ 1.3 mmol/l and no treatment, LDL ≤ 2.6 mmol/l and no treatment, hs-CRP ≤ 3.0 mg/l, and HOMA-IR ≤ 2.7 [12].

### Complete body composition analysis

Body composition was assessed using a multi-frequency bioelectric impedance analyzer (BIA): Inbody 770 Scanner (Inbody, Seoul, Korea). This electrical impedance analyzer measures the resistance of body tissues to the flow of an electrical signal sent through both feet and hands. The body composition analyzer was used to measure body fat mass (BFM), fat-free mass (FFM), body fat percentage (%), of the subjects following a standardized procedure according to guidelines. Participants were asked not to exercise vigorously, carry electrical appliances, drink extra fluids or food; measurements were taken in the morning and on an empty stomach, and participants urinated before analyzing their body composition for more accurate results.

### Assessment of dietary intake

Dietary intake was assessed using a 147-items semi-quantitative food frequency questionnaire (FFQ) with high validity and reliability [33]. All FFQs were completed by a trained nutritionist. Participants were asked to rate their consumption of each food consumed in the previous year, daily, weekly, or monthly. Finally, we converted portion sizes of foods to grams/day by using household measures [34], and then For the evaluation of macro- and micronutrient content, N4 software was used.

### Construction of the MIND diet score

To calculate the MIND diet scores, some components of the FFQ diet were used in this study. There are 15 dietary parameters in the original scoring method of the MIND diet; 10 of them were defined as brain-healthy food groups (green leafy vegetables, other vegetables, nuts, beans, whole grains, berries, fish, poultry, olive oil, and wine), and 5 were classified as brain-unhealthy food groups (red meats, butter and stick margarine, cheese, pastries and sweets, and fast/ fried food) [29]. In the present study, we used modified MIND diet scoring based on Iranian eating habits [35]. Wine consumption was not included because drinking is prohibited. Therefore, 14 other food groups were used in MIND scoring. We adjusted all these components to energy using the residual method. First, participants were classified based on tertile categories of dietary intake. Participants in the lowest tertile of brain-healthy food groups, including green leafy vegetables, other vegetables, olive oil, nuts, berries, beans, whole grains, fish, and poultry intake, were given the score of 0, those in the middle tertile were given the score of 0.5, and those in the highest tertile were given the score of 1. On the other hand, participants in the lowest tertile of brain-unhealthy food groups, including red meat, butter and stick margarine, cheese, pastries and sweets, and fast/fried food intake, were assigned the score of 1, and participants with the highest consumption of these food groups were given the score of 0. Individuals in the middle tertile of these components were assigned a score of 0.5. Finally, the total score was calculated by summing up all the scores of the dietary components. Therefore, the participants’ diet score ranged from 0 to 14 [36].

### Assessment of other variables

To evaluate the level of physical activity in this study, a validated International Physical Activity Questionnaire (IPAQ) was used, which measures a person’s total physical activity in the past week in MET-minutes / week. According to the IPAQ scoring protocol, physical activity levels were classified into three groups: lowly active, moderately active, and highly active [37]. General information, including participants’ age, education level, marital status, job, supplementation, and economic status, was collected using a demographic questionnaire.

### Statistical analysis

First of all, before statistical procedures, preliminary analyses have been done, such as assessing the normal distribution of variables. According to the histogram and the Kolmogorov-Smirnov test distributions, they were closer to normal. The chi-square test was used to evaluate the relationship between the MIND score and categorical variables, which have been shown by number and percentage. In addition, a one-way analysis of variance (ANOVA) was used to evaluate significant mean differences of continuous variables among MIND tertiles according to (tertile1: < 6, tertile2: 6–8, tertile3: > 8) which have been shown by mean ± standard deviation (SD). The Analysis of Covariance (ANCOVA) was applied to compare the adjusted mean difference of the demographic characteristics and dietary intakes of participants among tertiles of the MIND diet by controlling the effect of energy intake for the dietary intakes and further with age, physical activity, and BMI for demographic characteristics. In some analyses, BMI considers as collinear. Also, binary logistic regression was applied to assess the association between the MIND diet score and overweight/obesity phenotypes in the crude model, as well as by adjusting for confounding factors such as age, energy intake, BMI, physical activity, and marital status in the two models. According to binary logistic regression, the data is presented as Odds ratio (OR) and 95% confidence interval (CI). We evaluated the odds of being metabolically unhealthy (MUH) compared to the reference group (MH) across the MIND tertiles; the first tertile of the MIND score was considered the reference category. P-values < 0.05 were considered significant in this study. All statistical analyses were performed via the Statistical Package for Social Sciences (version 24; SPSS Inc., Chicago, IL, USA).

## Result

### Study population characteristics

229 women, including 63 MHOW/O (27.5%) and 166 MUHOW/O individuals (72.5%), with a mean age of 36.16 (8.33) years, were recruited in the study. The mean (± SD) height, weight, and BMI of participants were 161.36 (5.77) cm, 80.22 (12.14) kg, and 30.76 (4.23) kg/m^2^, respectively. Also, the mean (SD) of biochemical variables and inflammatory parameters, including FBS, TG, HDL, LDL, total cholesterol (TC), and hs-CRP, of participants were 86.99 (9.65), 120.06 (59.86), 46.20 (10.88), 94.77 (24.18), 185.61 (35.86), and 4.40 (4.57), respectively. In terms of economic status and education, most participants were moderate 184 (45.5%), bachelor and higher 189 (46.8%), respectively.

### Baseline characteristics of study population among tertiles of the MIND diet score

The general characteristics of study participants among the tertiles of the MIND diet score are shown in Table 1. According to the results, in crude mode, the variables FFM (P = 0.04) and TG (P = 0.02) were significant, while after controlling for potentially confounding variables (age, energy intake, physical activity, BMI) lost their significance mean difference (P > 0.05). After controlling for potential confounders, a significant difference was also observed for marital status distribution (P = 0.001). There were no significant differences in any other variables across tertiles of MIND diet scores (P > 0.05).

**Table 1:** Baseline characteristics of study participants categorized according to tertiles of the MIND diet score in obese and overweight women (n = 229)

	Tertiles of the MIND diet score				
Variables	T_1_ (n = 77) < 6	T_2_ (n = 82) 6–8	T_3_ (n = 70) 8<	P value	(P value)*
	Mean ± SD				
**Quantitative variable**					
**Demographic characteristic**					
Age (Y)	35.05 ± 8.08	37.07 ± 8.81	36.23 ± 8.40	0.32	0.28
PA (MET-min/week)	1013.80 ± 1651.44	1152.99 ± 18688.79	1722.12 ± 3125.71	0.17	0.26
**Anthropometry and body Composition**					
Weight (kg)	77.39 ± 11.00	81.45 ± 13.15	81.38 ± 10.72	0.05	0.41
Height (cm)	160.59 ± 5.55	161.43 ± 5.60	161.81 ± 6.19	0.41	0.94
BMI (kg/)	30.04 ± 4.16	31.17 ± 4.63	31.17 ± 3.77	0.16	0.47
BF (%)	40.78 ± 5.67	41.51 ± 5.06	41.23 ± 5.69	0.70	0.99
BFM (kg)	32.00 ± 8.18	34.10 ± 9.01	34.28 ± 7.23	0.16	0.62
FFM (kg)	45.33 ± 4.89	47.18 ± 5.98	47.38 ± 5.60	**0.04**	0.44
**Blood pressure**					
SBP (mmHg)	110.65 ± 13.41	112.96 ± 13.47	113.03 ± 13.99	0.48	0.67
DBP (mmHg)	78.08 ± 11.34	78.71 ± 9.00	77.84 ± 9.27	0.86	0.10
**Biochemical variables**					
Insulin (mIU/mL)	1.18 ± 0.24	1.23 ± 0.23	1.23 ± 0.21	0.40	0.96
TG (mmol/L)	1.19 ± 0.09	1.44 ± 0.08	1.56 ± 0.10	**0.02**	0.11
HDL-C (mmol/L)	1.19 ± 0.03	1.16 ± 0.03	1.24 ± 0.03	0.23	0.51
LDL-C (mmol/L)	2.43 ± 0.07	2.34 ± 0.06	2.52 ± 0.08	0.22	0.28
TC (mmo/l)	4.74 ± 0.10	4.64 ± 0.10	4.92 ± 0.12	0.23	0.52
HOMA index	3.24 ± 0.15	3.43 ± 0.14	3.47 ± 0.17	0.52	0.89
**Inflammatory parameter**					
hs-CRP (mg/L)	4.56 ± 0.53	4.00 ± 0.49	4.31 ± 0.62	0.74	0.66
**Categorical variables** ^*^					
**Education**				0.48	0.76
Illiterate	0 (0)	3 (100)	0 (0)		
Under diploma	7 (30.4)	10 (43.5)	6 (26.1)		
Diploma	33 (37.5)	32 (36.4)	23 (26.1)		
Bachelor and higher	36 (31.9)	48 (42.5)	29 (25.7)		
**Marital status**				0.09	**0.001**
Single	15 (30.6)	13 (26.5)	21 (42.9)		
Married	60 (33.7)	69 (38.8)	49 (27.5)		
**Job**				0.67	0.53
non-employed	41 (31.1)	49 (37.1)	42 (31.8)		
Employed	33 (36.3)	33 (36.3)	25 (27.5)		
**Supplementation**				0.67	0.37
Yes	29 (28.4)	39(38.2)	34 (33.3)		
No	24 (34.3)	26 (37.1)	20 (28.6)		
**Economic status**				0.28	0.94
Poor	17 (28.8)	26 (44.1)	16 (27.1)		
Moderate	39 (40.2)	33 (34)	25 (25.8)		
Good	17 (28.8)	20 (33.9)	22 (37.3)		

BF%; body fat percentage; BFM: body fat mass; BMI: body mass index; DBP: diastolic blood pressure; FFM: fat free mass; HDL-C: high density lipoprotein cholesterol; HOMA; homeostatic model assessment; hs-CRP: high-sensitivity C-reactive protein; LDL-C: low density lipoprotein cholesterol; MIND: Mediterranean-DASH Intervention for Neurodegenerative Delay; PA: physical activity; SBP: systolic blood pressure; SD: Standard Deviation; T: tertile; TC: total cholesterol; TG: triglyceride

P value: ANOVA test was used

(P value)*: ANCOVA was performed to adjusted potential confounding factors (age, energy intake, Physical activity, BMI), BMI consider as collinear variable for anthropometrics and body composition variables

Chi-square was used for categorical variables

P-values < 0.05 were considered as significant

Values are represented as means ± SD.

*categorical variables: N(%)

### Dietary intakes of study subjects according to tertiles of the MIND diet score

The dietary intake of participants across tertiles of the MIND-diet score is shown in Table 2. As shown, after adjustment with the energy intake, there was a significant mean difference among some of the food groups, and participants in the highest tertiles of this score had higher intakes of all kinds of green leafy vegetables, other vegetables, olive oil, nuts, beans, whole grains, fish, and poultry (P < 0.001), berries, (P = 0.003), but lower intakes of butter and stick margarine, pastries and sweets (P < 0.001), and fast/fried food intake (P = 0.01). Also, after adjusting for energy intake the significant mean difference dietary intakes of macronutrients including carbohydrates (P = 0.01), total Fat, protein (P < 0.001), and micronutrients including iron, zinc, magnesium, vitamin A, vitamin k, niacin, pantothenic acid, vitamin B6, folate (P < 0.001), monounsaturated fatty acid (MUFA), calcium, vitamin D (P = 0.008), riboflavin, total fiber (P = 0.004), polyunsaturated fatty acid (PUFA) (P = 0.03), saturated fatty acid (SFA) (P = 0.001), of the study participants, were observed among tertiles of MIND diet scores.

**Table 2:** Dietary intakes of study subjects according to tertiles of the MIND diet score in obese and overweight women (n = 229)

	Tertiles of the MIND diet score						
Variables			T_1_ (n = 77) < 6	T_2_ (n = 82) 6–8	T_3_ (n = 70) 8<	P value	(P value)*
	Mean ± SD						
**Food groups**							
Whole grains (g/d)			438.45 ± 165.54	483.45 ± 216.22	526.72 ± 283.71	**0.006**	0.64
Olive oil (g/d)			1.65 ± 0.71	2.01 ± 0.80	2.37 ± 0.77	**< 0.001**	**< 0.001**
Berries (g/d)			1.90 ± 3.91	4.45 ± 8.34	5.02 ± 8.61	**0.001**	**0.003**
Other vegetables (g/d)			283.66 ± 214.17	498.52 ± 617.57	706.15 ± 589.23	**< 0.001**	**< 0.001**
Green leafy vegetables (g/d)			36.08 ± 29.60	72.11 ± 70.87	93.94 ± 56.93	**< 0.001**	**< 0.001**
Beans (g/d)			30.27 ± 20.54	46.26 ± 32.38	68.40 ± 51.49	**< 0.001**	**< 0.001**
Nuts (g/d)			0.40 ± 0.42	0.46 ± 0.38	0.63 ± 0.38	**< 0.001**	**0.001**
Poultry (g/d)			23.93 ± 19.81	35.09 ± 36.12	51.06 ± 49.27	**< 0.001**	**< 0.001**
Fish			6.29 ± 6.56	12.33 ± 13.76	14.96 ± 13.47	**< 0.001**	**< 0.001**
Butter and stick margarine (g/d)			16.76 ± 22.59	14.91 ± 22.36	9.24 ± 15.60	**0.009**	**< 0.001**
Cheese			31.79 ± 25.85	29.78 ± 22.92	29.18 ± 36.44	0.73	0.25
Red meat			19.00 ± 16.15	24.56 ± 25.56	21.98 ± 19.45	0.08	0.26
Pastries and sweets			65.68 ± 73.01	58.05 ± 89.11	39.38 ± 61.68	**0.01**	**< 0.001**
Fast/ fried food			26.61 ± 29.40	26.72 ± 27.59	23.35 ± 27.90	0.55	**0.01**
**Nutrients**							
Energy (kcal)			2467.25 ± 762.26	2685.67 ± 807.53	2740.67 ± 697.82	0.06	-
Carbohydrates (g/d)			342.06 ± 14.00	379.06 ± 13.47	404.79 ± 14.58	**0.008**	**0.01**
Total Fat (g/d)			95.20 ± 3.87	98.27 ± 3.73	89.85 ± 4.03	0.30	**< 0.001**
Protein (g/d)			75.93 ± 2.99	91.23 ± 2.88	101.25 ± 3.12	**< 0.001**	**< 0.001**
Total fiber (g/d)			39.40 ± 2.09	44.83 ± 2.01	51.28 ± 2.18	**0.001**	**0.004**
MUFA (g/d)			31.72 ± 1.38	31.95 ± 1.33	30.02 ± 1.44	0.57	**0.007**
PUFA (g/d)			20.72 ± 1.08	20.30 ± 1.04	18.98 ± 1.13	0.51	**0.03**
SFA (g/d)			28.57 ± 1.29	28.88 ± 1.24	26.80 ± 1.35	0.48	**0.001**
Trans fat			0.001 ± 0.00	0.001 ± 0.00	0.002 ± 0.00	0.22	0.28
Iron (mg/d)			16.45 ± 0.66	18.91 ± 0.64	21.02 ± 0.69	**< 0.001**	**< 0.001**
Zinc (mg/d)			11.47 ± 0.47	13.55 ± 0.45	14.36 ± 0.49	**< 0.001**	**< 0.001**
Calcium (mg/d)			1021.06 ± 47.20	1221.55 ± 45.44	1272.74 ± 49.18	**0.001**	**0.008**
Magnesium (mg/d)			396.54 ± 16.14	479.54 ± 15.54	526.45 ± 16.82	**< 0.001**	**< 0.001**
Vitamin C (mg/d)			158.81 ± 14.93	205.52 ± 14.37	228.59 ± 15.56	**0.005**	0.05
Vitamin E (mg/d)			16.43 ± 1.05	18.04 ± 1.01	16.61 ± 1.09	0.48	0.47
Vitamin A (mg/d)			617.59 ± 45.52	828.93 ± 43.82	967.91 ± 47.43	**< 0.001**	**< 0.001**
Vitamin K (mg/d)			129.59 ± 22.70	227.97 ± 21.85	304.43 ± 23.65	**< 0.001**	**< 0.001**
Vitamin D (ug/d)			1.48 ± 0.18	2.35 ± 0.17	2.20 ± 0.19	**0.002**	**0.008**
Thiamin (mg/d)			1.89 ± 0.07	2.09 ± 0.07	2.23 ± 0.07	**0.008**	0.05
Riboflavin (mg/d)			1.92 ± 0.09	2.39 ± 0.09	2.35 ± 0.09	**< 0.001**	**0.004**
Niacin (mg/d)			21.57 ± 0.96	25.61 ± 0.93	28.90 ± 1.00	**< 0.001**	**< 0.001**
Pantothenic acid (mg/d)			5.43 ± 0.27	6.90 ± 0.26	7.32 ± 0.28	**< 0.001**	**< 0.001**
Vitamin B6 (mg/d)			1.82 ± 0.07	2.20 ± 0.07	2.55 ± 0.07	**< 0.001**	**< 0.001**
Folate (mcg/d)			530.80 ± 19.17	619.14 ± 18.46	681.62 ± 19.98	**< 0.001**	**< 0.001**
Vitamin B12 (mcg/d)			3.82 ± 0.28	4.84 ± 0.27	4.78 ± 0.29	**0.01**	0.11

MUFA; monounsaturated fatty acid; PUFA: polyunsaturated fatty acid; SD: Standard Deviation; SFA: saturated fatty acid; T: tertile

P value: ANOVA test was used

(P value)*: ANCOVA was performed to adjusted potential confounding factors (energy intake).

Values are represented as means ± SD.

P-values < 0.05 were considered as significant

### The association between MIND diet score classifications with overweight/obesity phenotypes

The association between MIND diet score with overweight/obesity phenotypes and the ORs (95% CI) of MUHOW/O comparison to MHOW/O across tertile categories of MIND diet score are shown in Table 3. In the crude model, there was no significant association between overweight/obesity phenotypes and the MIND score (OR: 1.63, 95% CI: 0.79–3.33, P-value = 0.18), and the odds of MUH relative to MH had higher nonsignificant in the highest tertiles of the MIND diet score (P-trend = 0.26). In the age, energy intake, BMI, and physical activity-adjusted model 1, there was no significant association observed between overweight/obesity phenotypes with tertile 2 (T2) (OR: 2.01, 95% CI: 0.86–4.17, P-value = 0.10), T3 (OR: 1.89, 95% CI: 0.86–4.17, P-value = 0.11) of MIND score, and only the odds of MUH relative to MH with a marginal significant decreasing trend was observed from the second to the third tertile (1.89 vs. 2.01) (P-trend = 0.06). Also, after additional adjustment for marital status, the nonsignificant association between overweight/obesity phenotypes with tertile 2 (T2) (OR: 2.13, 95% CI: 0.89−5.10, P-value = 0.08), T3 (OR: 1.87, 95% CI: 0.83−4.23, P-value = 0.12) of MIND score remained, and the odds of MUH relative to MH with a significant decreasing trend was observed with increasing tertiles (P-trend = 0.04).

**Table 3:** Odds ratios (and 95% CIs) for overweight/obesity phenotypes (in MUHOW/O comparison to MHOW/O) across tertiles of MIND score in obese and overweight women (n = 229)

Models			Tertiles of the MIND diet score				
	T_1_ (n = 77) 6<	T_2_ (n = 82) 6–8		P-value	T_3_ (n = 70) 8<	P-value	P- trend
			OR (95%CI)		OR (95%CI)		
**Crude**	Ref	1.52 (0.73−3.14)		0.25	1.63 (0.79−3.33)	0.18	0.26
**Model 1**	Ref	2.01 (0.86−4.71)		0.10	1.89 (0.86−4.17)	0.11	**0.06**
**Model 2**	Ref	2.13 (0.89−5.10)		0.08	1.87 (0.83−4.23)	0.12	**0.04**

CI: Confidence Interval; OR: odds ratio; T: tertile

P-values are reported base on the Binary logistic regression test

p-values < 0.05 were considered as significant and 0.05, 0.06, and 0.07 was considered marginally significant

Metabolically healthy is a reference group

Model 1: Adjusted for age, energy intake, BMI and physical activity

Model 2: Model 1 further adjustment with Marital status

## Discussion

To our knowledge, this is the first study investigating the association between the MIND diet and MH and MUH phenotypes. In this study, we did not find a significant association between this score and MH/MUH phenotypes, but we did see a decreasing trend in MUH odds compared to MH by moving to the top tertile.

Due to the lack of studies on the association between the MIND diet and obesity phenotypes, studies on the association between the DASH diet and the Mediterranean diet, because the MIND diet is a combination of the DASH and the Mediterranean diet [29], and obesity phenotypes and its components according to the Karelis criteria, will be reviewed. Some diet component such as amount of refined grains, fast foods [38], trans fatty acids, cholesterol [39], fruits, vegetables [40], fiber [41] intake, and each macronutrient may have different effects on the risk factors causing MUH and risk of MUH. Therefore, to examine the results regarding the association between this score and the risk of MUH, the dietary composition should be considered.

Some studies have shown a link between the DASH diet and obesity phenotypes [18, 20, 21, 42, 43]. In a study done by Park et al. in men and women under the age of 45 same as preset study, was found that a high Mediterranean diet score was associated with a higher odds of healthy obesity phenotype [20]. Phillips et al. found a significant association between DASH score and metabolic health in obese men and women aged 45–74 years [21]. In a randomized controlled trial study by Azadbakht et al., stated that more adherence to the DASH diet is inversely associated with MUH [44], also Mirzababaei et al. found various associations betrween major dietary patterns and MUH/MH [12]. The reasons for not seeing these associations, but seeing the significant trends between the MIND diet and obesity phenotypes, can be due to differences in study designs such as obesity status of the study population, for example, in the normal weight population or the obese and overweight population), age, gender (female and male).

The positive effect of the DASH diet on insulin-resistant as a component of Karelis criteria can be due to the good intake of whole grains, fruits, vegetables, nuts, and seeds, which makes the diet rich in fiber and antioxidants, and Magnesium, all of which play a role in reducing inflammation [45]. On the other hand, whole grains due to their high fiber content, cause slow absorption of carbohydrates in the gastrointestinal tract and decrease glycemic index, which ultimately reduces the rate of hyperglycemia and blood insulin [46]. Also, high levels of potassium, magnesium, vitamin C, and phytochemicals in this diet are associated with decreased IR, which is an effective component in causing MUH, in obesity and metabolic syndrome [47]. In addition, high fiber intake reduces TG, blood pressure and fasting blood sugar [48]. Since the MIND diet is based on the DASH diet, these proposed mechanisms can also be valid for the MIND diet [29]. As in our study, the intake of items mentioned was higher in the MH group and the upper tertile of MIND than in the lower tertile. whilst the intake of whole grains was not higher in MH group, also it’ intake was decreased with increasing in tertiles, this can be one of the reasons for not observing the association between the MIND diet and MH/MUH phenotypes but causes the significant trends. The diet pattern with high consumption of margarine, snacks, and sweets has a positive relationship with IR, which is in contrast to the diet pattern of the DASH diet [49]. So, following the DASH diet reduces IR, which is one of the factors contributing to the unhealthy obesity phenotype. The Mediterranean diet is rich in polyphenolic compounds, antioxidants, fiber, and magnesium, all of which reduce IR [50], which is an important factor in metabolic disorders in overweight and obese people. Good intake of MUFA is due to the relatively good consumption of olive oil in the Mediterranean diet, which is associated with lower TG and increased HDL_C in the blood [51], which affect metabolic health. Magnesium is associated with a reduced risk of metabolic syndrome [52] and type 2 diabetes [53]. As in our study, the consumption of berries, beans, vegetables, nuts, and olive oil in the upper tertile is higher than in the lower tertile. Also, it was found that in the upper tertile of the MIND diet, which has a lower risk for MUH, there is a good intake of fiber and Magnesium, which confirms the positive effect of mg and fiber (either individually or synergistically) on metabolic health.

The DASH and Mediterranean diets have a higher fiber content than the MIND diet because not all fruits are included in the MIND diet [54], also, the consumption of beans has generally decreased due to the nutritional transition that has occurred in Asian countries [55]. Differences in fiber consumption can be a reason that explains why only significant trends are observed in the present study. Another reason could be that the MIND diet only focuses on cheese and other low-fat dairy products do not receive much attention, unlike the Mediterranean diet [54]. In general, the MIND diet (DASH + Mediterranean diet), through its pattern and dietary content, may have positive effects on maintaining healthy metabolism in obese people or weight control.

Strengths of this study, we can mention to the 168-item FFQ questionnaire validated according to the Iranian diet, also examining physical activity status, removing drug users from the study described in the method section.

One of the limitations of this study is it’s cross-sectional design due to it’s limitation to show cause and effect relationships and it’s done only on women. Also score the Mediterranean diet in non-Mediterranean populations is difficult. Comparing our results with the results of other studies in the world, due to racial, gender, and other differences.

## Conclusion

In conclusion, no significant associations were found between adherence to MIND diet with MUH, and only a significant downward trend in the odds of MUH was observed with increasing tertiles. More longitudinal studies are needed to confirm these findings.

## Data Availability

The data that support the findings of this study are available from Khadijeh Mirzaei but restrictions apply to the availability of these data, which were used under license for the current study, and so are not publicly available. Data are however available from the authors upon reasonable request and with permission of Khadijeh Mirzaei.

## List of abbreviations

ANCOVA: Analysis of Covariance
BF%: Body fat percentage
BFM: Body fat mass
BIA: Bioelectrical Impedance Analyzer
BMI: Body mass index
CHOD-PAP: Phenol 4-Aminoantipyrine Peroxidase
CIs: Confidence intervals
CVDs: Cardiovascular diseases
DBP: Diastolic blood pressure
EDTA: Ethylenediaminetetraacetic acid
FBS: Fasting blood sugar
FFM: Fat free mass
GOD/PAP: Glucose oxidase phenol 4-Aminoantipyrine Peroxidase
HDL-C: High density lipoprotein cholesterol
HOMA: Homeostatic model assessment
hs-CRP: High-sensitivity C-reactive protein
IR: Insulin resistance
IPAQ: International Physical Activity Questionnaire
LDL-C: Low density lipoprotein cholesterol
MH: Metabolically healthy
MHOW/O: Metabolically healthy overweight/obesity
MIND: Mediterranean-DASH Intervention for Neurodegenerative
MUFA: Monounsaturated fatty acid
MUH: Metabolically unhealthy
MUHOW/O: Metabolically unhealthy overweight/obesity
PA: Physical activity
PUFA: Polyunsaturated fatty acid
SBP: Systolic blood pressure
SDs: Standard Deviations
SFA: Saturated fatty acid
T: Tertile
T2D: Type 2 diabetes
TC: Total cholesterol
TG: Triglyceride
